# Genomic, RNA, and ecological divergences of the *Revolver *transposon-like multi-gene family in Triticeae

**DOI:** 10.1186/1471-2148-11-269

**Published:** 2011-09-25

**Authors:** Motonori Tomita, Asuka Okutani, Avigdor Beiles, Eviatar Nevo

**Affiliations:** 1 Molecular Genetics Laboratory, Faculty of Agriculture, Tottori University, Tottori 680-8553, Japan; 2 Institute of Evolution, University of Haifa, Mount Carmel, Haifa 31905, Israel

## Abstract

**Background:**

*Revolver *is a newly discovered multi-gene family of transposable elements in the Triticeae genome. *Revolver *encompasses 2929 to 3041 bp, has 20 bp of terminal inverted repeated sequences at both ends, and contains a transcriptionally active gene encoding a DNA-binding-like protein. A putative TATA box is located at base 221, with a cap site at base 261 and a possible polyadenylation signal AATAAA at base 2918. *Revolver *shows considerable quantitative variation in wheat and its relatives.

**Results:**

*Revolver *cDNAs varied between 395 and 2,182 bp in length. The first exon exhibited length variation, but the second and third exons were almost identical. These variants in the *Revolver *family shared the downstream region of the second intron, but varied structurally at the 5' first exon. There were 58 clones, which showed partial homology to *Revolver*, among 440,000 expressed sequence tagged (EST) clones sourced from Triticeae. In these *Revolver *homologues with lengths of 360-744 bp, the portion after the 2nd exon was conserved (65-79% homology), but the 1st exon sequences had mutually low homology, with mutations classified into 12 types, and did not have EST sequences with open reading frames (ORFs). By PCR with the 3'-flanking region of a typical genomic clone of *Revolver*-2 used as a single primer, rye chromosomes 1R and 5R could be simultaneously identified. Extensive eco-geographic diversity and divergence was observed among 161 genotypes of the single species *Triticum dicoccoides *collected from 18 populations in Israel with varying exposures to abiotic and biotic stresses (soil, temperature, altitude, water availability, and pathogens).

**Conclusions:**

On the base of existing differences between *Revolver *variants, the molecular markers that can distinguish different rye chromosomes were developed. Eco-geographic diversification of wild emmer *T. dicoccoides *in Israel and high *Revolver *copy numbers are associated with higher rainfall and biotic stresses. The remarkable quantitative differences among copy numbers of *Revolver *in the same species from different ecosystems suggest strong amplification activity within the last 10,000 years. It is the interesting finding because the majority of Triticeae high-copy transposable elements seem to be inactive at the recent time except for *BARE-*1 element in *Hordeum *and the fact might be interesting to perceive the processes of plant adaptive evolution.

## Background

In the higher plants, only a small percentage of the genome is required for maintenance of life [1,2], and transposable elements and the sequences derived from them are scattered in the other highly repetitive DNA regions [3-6]. The transposable elements are classified into the class I transposable elements (retrotransposons), which use a transcript as a template and transfer replicatively, and class II transposable elements (transposons), which transfer DNA by the cut-and-paste mechanism. It is believed that class I long terminal repeat (LTR) retrotransposons and class II miniature inverted repeat transposable elements (MITEs) are major components of the plant genome [7-10]. Such transfer factors can be used as the source of mutations for DNA marker development or gene functional analysis; the transposable elements with a high number of copies can become the entry points for PCR during DNA marker development, and the transposable elements that cause gene disruption enable tagging of genes and contribute to functional genome research [11,12]. However, the sequences of regions of repetitive DNA other than the known transposable elements [13,14] are still undetermined, while in the region of repetitive DNA regarded as junk DNA, it has been found that the RNA genes that perform epigenetic regulation of gene expression are also scattered [15-17]. Investigation of the unknown factors in repetitive DNA regions is also important as a key to understanding the mechanisms of genome control and phenotypic expression.

The first authors recently reported a new transposon-like gene, named *Revolver*, in Triticeae [18,19]. A part of the reiterated sequence (89 bp) specific to the rye genome was cloned by the genome subtraction technique, which deducts the sequence in common with bread wheat from the rye genome [20]. In order to determine the entire structure of this reiterated sequence, the λ FixII genomic library of the rye-inbred line was screened by using 89 bp of the repetitive clone as a probe, and the base sequence of a region of about 21.6 kb was decoded in three lambda clones. As a result, the insertion-type consensus sequence (92% homology) with a full-length sequence of 3,041 bp sandwiched between 20 bp of specific terminal inverted repeat (TIR) sequence was determined and shown to be similar to the class II transposable elements [18,21-26]. *Revolver *contains one gene that codes for an open reading frame (ORF) of a deduced 139 amino acid residue that is actively transcribed into mRNA. The sequence of the 20 bp inverted repeat sequence at both ends is different from that of the known transposons represented by *hAT*, CACTA, and *Mutator*, and 10 bp of the tandemly repeated sequence is also repeated in the subterminal region.

In a genomic DNA clones of rye that show homology to *Revolver*, great structural variation in the region ranging from the first exon to the first intron had arisen and four sequences thought to be nonautonomous factors for *Revolver *were found. *Revolver *produces 0.7 kb of mRNA and is conserved in Triticeae. A putative TATA box is located at base 221, with a cap site at base 261 and a possible polyadenylation signal AATAAA at base 2918 [18]. On the other hand, the full length of the nonautonomic factor is 2665 to 4269 bp. The nonautonomic factor has a 37-149-bp homologous region upstream from the transcription start site containing a TIR at the 5' end and a 1294-2112-bp region covering from around the second exon to the 3' end on the 3' side. A region of 549-2007 bps located between them (equivalent to the region from the first exon to the first intron) is destroyed. In rye and barley, the nonautonomic factors sharing each end with *Revolver *are considered to exist within it [19].

The number of copies in Triticeae was computed by Southern blot analysis and slot blotting techniques by using cDNA of *Revolve*r as the probe. Consequently, extremely high numbers of *Revolver *(20,000 copies) have been shown to exist in *Dasypyrum villosum *and *Secale *sp., as well as in diploid species such as *Triticum monococcum*, which is the ancestor of bread wheat and tetraploid species such as emmer wheat, with around 10,000 copies. However, the number of copies is extremely low in hexaploid bread wheat. There has been a large quantitative change during the evolution of Triticeae resulting in amplification of *Revolver *in some species and its disappearance in bread wheat [18]. The considerable quantitative variability of *Revolver *among the wheat-related species strongly indicates its propagation or differential loss, activity, and diversity in recent evolutionary times.

In this paper, the structural divergence of *Revolver *in genomic DNA and RNA was analyzed, and length variants of the *Revolver *family were used as chromosome tags to search the publicly disclosed expressed sequence tag (EST) database of the 440,000 Triticeae EST sequences for *Revolver *homologues. Moreover, we examined the quantitative variations of *Revolver *in 161 *T. dicoccoides *accessions [27] representing 18 populations collected from various locations in Israel, which encompass a wide range of ecologic conditions of soil, temperature, altitude, and water availability, to determine the effects of ecologic stress on quantitative variation.

## Results and Discussion

### Structural diversity of *Revolver *mRNA

**Figure 1:**
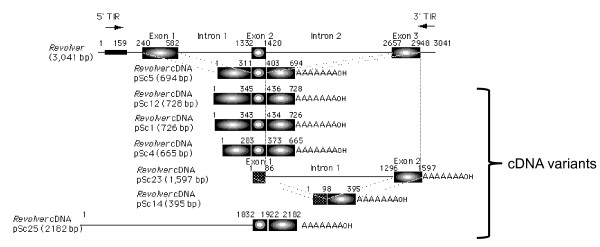
**Multiple classes of *Revolver *mRNA**. The structure of several *Revolver *cDNA clones (AB124645, 124665-124669) isolated from rye in comparison with *Revolver *genomic sequence.

*Revolver*, encompassing a 3,041 bp sequence, has 20 bp of TIR sequences and contains a transcriptionally-active gene, consisting of three exons (342 bp, 88 bp, and 296 bp) and two introns (750 bp and 1,237 bp) (Figure 1), and encodes a DNA-binding-like protein [18]. Fifty cDNA clones of the *Revolver *family were obtained from self-fertile rye (homozygous genotype), and a structural analysis was performed. The total lengths of typical *Revolver *cDNAs were 665 to 723 bp in 40 clones out of 50 cDNAs, and they were classified into three subfamilies (I, II, and III) in which the regions of the second and third exons were almost identical, while the region of the first exon exhibited a low homology of 60% among the subfamilies because of duplication or deletion (Figure 1 and Additional file 1). The homologies in the subfamilies were 89% ( I), 97% ( II), and 93% ( III), and the homologies between the subfamilies were 75% for subfamily I and subfamily III, 80% for subfamily I and subfamily III, and 76% for subfamily I and subfamily III. Comparing sequences between the exons, the second exon (89 to 92 bp) and the third exon (293 bp) exhibited high homologies of 91 to 95% between the different subfamilies. In contrast, the first exon exhibited high homology within the subfamilies (I: 98%, II: 99%, and III: 99%); but the homologies between subfamilies were low (63% between I and II, 64% between I and III, and 67% between II and III). In the first exon, partial deletions and mutations of different lengths were found in the base sequences of the three classes. Thus, the classification of the cDNA corresponds to the structural divergence of the first exon. Because repetitive sequence units composed of 8 to 14 bp are present in the same direction in the first exon, a nonhomologous recombination between alleles might have caused various structural mutations.

Moreover, cDNAs exhibiting completely different structures at the first exon were found in 50 cDNA clones from self-fertile rye (Figure 1). Among them, five cDNAs had a total length of 1,597 bp and contained the second intron (1,210 bp) and the third exon (301 bp) of *Revolver*, but at the 5' terminus, they had an 86 bp sequence that was not observed in *Revolver*. On the other hand, four cDNA clones had a total length of 395 bp and lacked the second intron compared to the cDNA clones of 1,597 bp described above. Meanwhile, another cDNA clone (total length of 2,182 bp) had the second exon (90 bp) and the third exon (260 bp) of *Revolver*, but the region corresponding to the first exon was extremely long and had no homology with the other cDNA clones. As stated above, the members of the *Revolver *family, having a common structure downstream of the second intron, may easily undergo various structural changes at the 5' first exon and then become divergent and show different lengths. Such structural diversities were found in the first exon of the *Revolver *genes that were obtained from the self-fertile pure rye strains, and these structural diversities may be useful for development of DNA markers in the rye genome.

**Figure 2:**
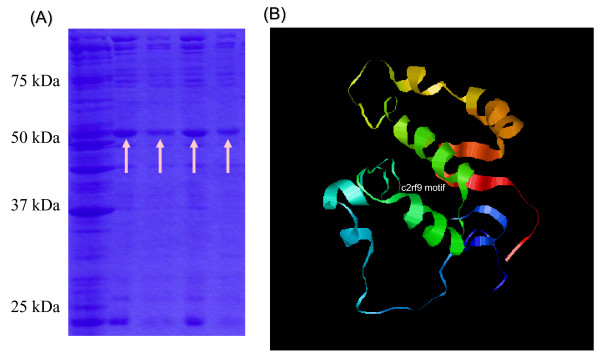
***Revolver *Protein**. (**A**) The *Revolver *ORF was integrated into the pET-32 vector, and the *Revolver*-encoded protein fused with thioredoxin was produced in *E. coli*. The molecular weight of the fused protein was 50,000. (**B**) The protein structure encoded by *Revolver *was predicted by the Protein Folding Recognition program Robetta.

Furthermore, *Revolver *cDNAs were recovered from *S. silvestre, D. villosum, T. monococcum*, and *Aegilops tauschii *after isolation by RT-PCR with 22-mer primers used at both ends. The majority belonged to subfamilies I (47%) or II (27%) of *S. cereale*, indicating that two major subfamilies were conserved within the *Triticum *species. Among the *Revolver *mRNAs, subfamily I contained a single ORF encoding 139 amino acid residues [18], which was conserved in the *Triticum *species with 98% homology. The *Revolver *ORF was integrated into the pET-32 vector, and the encoded protein fused with thioredoxin was produced in *Escherichia coli *(Figure 2A). The molecular weight of the fused protein was about 50,000, and that after treatment with Factor Xa was about 30,000 (Superdex 75 gel filtration). The protein structure encoded by *Revolver *was predicted by the Protein Folding Recognition program Robetta. As shown in Figure 2B, the *Revolver *ORF includes the c2rf9 motif of a kinase domain.

### Expressed sequence tag homologues of *Revolver*

**Figure 3:**
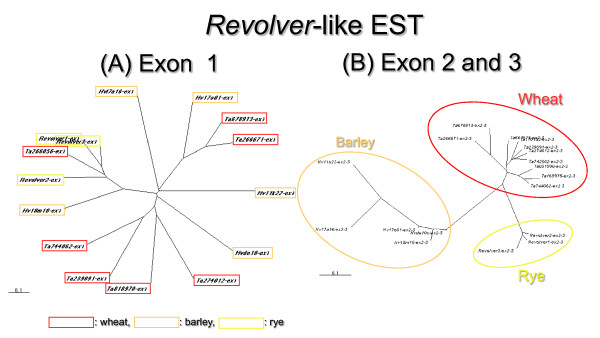
**Neighbor-joining tree of authentic *Revolver *cDNAs and *Revolver*-like Triticeae ESTs**. Two phylogeneric trees were constructed on the exon 1 region **(A)**, and on the exon 2 and exon 3 regions **(B)**, respectively. *Revolver*-like Triticeae ESTs were diverged into at least 12 types with regardless to species on the basis of mutations in the exon 1 region. On the other hand, the phylogeneric tree on the exon 2 and exon 3 regions showed three major clusters according to wheat, rye and barley species.

**Figure 4:**
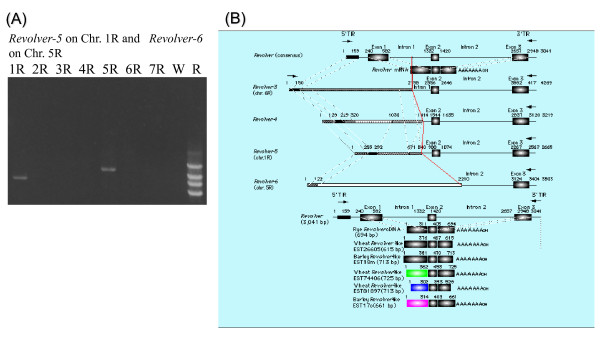
**Genomic divergences of the *Revolver *gene family offer chromosome tags**. (**A**) PCR with the 3'-flanking region of a typical *Revolver *(*Revolver*-2) used as a single primer amplified four DNA fragments (2.3 kb, 2.8 kb, 3.3 kb, and 4.3 kb) from the rye genome, but produced no fragments from the wheat genome. With this primer, rye chromosomes 1R and 5R can be identified in the wheat genome. (**B**) Each DNA fragment derived from the chromosome addition lines of 1R, 5R, and 6R (AB646252-646254) was a nonautonomous element of *Revolver*, with its second intron and the downstream region, but with considerable structural changes at the 5' end. Of the 440,000 Triticeae EST clones, only two clones, which were from *T. aestivum *(615 bp) and *H. vulgare *(713 bp), showed similarity with *Revolver *cDNA across its entire length. Other EST clones had low homology in the 5' exon 1 regions.

In this study, we searched the publicly disclosed EST database for *Revolver *homologues. Of the 440,000 Triticeae EST clones in GenBank having mainly *T. aestivum *as their source, 63 clones showed partial homology with *Revolver *(46 *T. aestivum*, 6 *S. cereale*, 4 *T. monococcum*, 2 *Ae. speltoides*, and 5 *H. vulgare*) (Figure 3). These *Revolver *homologues were about 360-744 bp in length. Only two clones, which were from *T. aestivum *(615 bp) and *H. vulgare *(713 bp), showed similarity with *Revolver *cDNA across its entire length (Figure 4B). Sequences downstream of exon 2 were conserved (about 65-79% identity) in other EST clones (Figure 3B), although they had either low homology in the 5' regions containing exon 1 (Figure 3A), or were missing in the upstream sequence. *S. cereale*, Einkorn wheats, and *Ae. tauschii*, all of which showed high levels of *Revolver *transcription by northern blotting, have cloned cDNA from the *Revolver *ORF coding for the 139 amino acids (Class I) together with 2 cDNAs differing in exon 1 sequences (Class II, III). The 615-bp EST from *T. aestivum*, which had the highest sequence similarity to *Revolver*, was close to Class II (Figures 3A and 4B); however, the exon 1 region was only 60% homologous. Other EST clones containing the exon 1 region matched none of the three classes and were very different from each other (Figures 3A and 4B). *Revolver*-like EST clones from *T. aestivum *were classified into at least 12 types on the basis of mutations in the exon 1 region, and these EST clones exhibited large genetic variation (Figure 3A). Exon 2 and exon 3 regions showed three major clusters according to wheat, rye and barley species (Figure 3B). Thus, *Revolver*-like transcripts do exist in *T. aestivum*, although their proportion among total EST clones is extremely small, and given that they include variants with large mutations, no true *Revolver *genes were present. We hypothesize that *Revolver *was lost in *T. aestivum *because it lost its ability to be reinserted. On the other hand, no *Revolver *homologues were found in the *Oryzeae *EST database. In addition, *Revolver *was not detected in the rice genome by Southern blot analysis.

### Development of chromosomal markers with *Revolver*

A typical genomic clone of *Revolver *(*Revolver*-2 [18]) was located in this study on the rye 7R chromosome because 492 bp of DNA was amplified only from the rye 7R chromosome addition line by PCR using the 5'-flanking region of *Revolver*-2, 5'-GCCTTTCGGCCTTCCTCTCAGGCGG-3', and its internal sequence of 5'-GTACTTGGCATCGGTAGATGTTCGG-3', as the primers. PCR was performed then with the 3'-flanking region derived from the typical genomic clone of *Revolver*-2 as a single primer, and 4 DNA fragments (2.3 kb, 2.8 kb, 3.3 kb, and 4.3 kb) were amplified from the rye genome, but nothing was amplified from the wheat genome (Figure 4A). Furthermore, when PCR was performed with the same primer and genomic DNA from rye chromosome addition lines as a template, DNA fragments of 2.8 kb, 3.6 kb, and 4.3 kb were amplified from the 1R, 5R, and 6R chromosome addition lines, respectively (Figure 4B). PCR amplification with this single primer identified rye chromosomes 1R and 5R simultaneously. Each DNA fragment derived from the chromosome addition lines and four types of DNAs amplified from the rye genome were sequenced (Additional file 2); and the variants were shown to have the downstream region of the second intron, but they had structural modifications at the 5' first exon region as in the cDNAs (Figure 4B). Such a difference in length in *Revolver *allows the development of rye chromosome markers.

*Revolver*-3 [18] comprises a total length of 4,269 bp, and at the 3' end it has a region of 2,112 bp from the middle of the first intron of *Revolver *through the third exon and reaches the 3'-terminal region (Figure 4B). In this study, *Revolver*-3 was shown to be localized on the 6R chromosome because it was amplified with the 3'-flanking region primer of *Revolver*-2 only from the rye 6R chromosome addition line. Furthermore, *Revolver*-5 located on the rye 1R chromosome had a total length of 2,665 bp, while at the 3' side it had a region of 1,826 bp extending from immediately before the second exon to the 3' terminus of *Revolver *(Figure 4A and 4B). At its 5' side, the region homologous to *Revolver *is limited to only 37 bp at the terminus; but a region of about 670 bp is homologous to *Revolver-*4 [18], which consists of 3,219 bp, and at the 3' end it has a region of 1,806 bp ranging from immediately before the second exon to the 3' terminus of *Revolver *(Figure 4B). Finally, *Revolver*-6 located on the rye 5R chromosome (Figure 4A) had a total length of 3,503 bp, and at the 3' side, it had a region of 1,294 bp from the middle of the second intron to the 3' terminus of *Revolver*. However, at the 5' side, there was no region homologous to *Revolver*, and the 121 bp sequence at the 5' terminus was homologous to *Revolver*-4 and *Revolver*-5 (Figure 4B).

As mentioned above, the members of the *Revolver *family showed considerable length variation which was attributed to structural changes in the first exon. Such a divergence in length is also found in some transposons, the CACTA family [28], the *Mutator *family [29], and the MITE family [10], but no homology was detected between *Revolver *and any of these. If the *Revolver *family is a transposable element, these variants are assumed to be nonautonomous elements. *Revolver *showed a variability that was considerably larger than the others. With PCR primers comprising the sequences flanking the length variants of *Revolver *scattered in the genome, the chromosome on which each *Revolver *is located can be determined or tagged, and such PCR primers can be utilized for detection and identification of the chromosomes.

### Quantitative variation of *Revolver *in wild Emmer wheat of Israel

**Figure 5:**
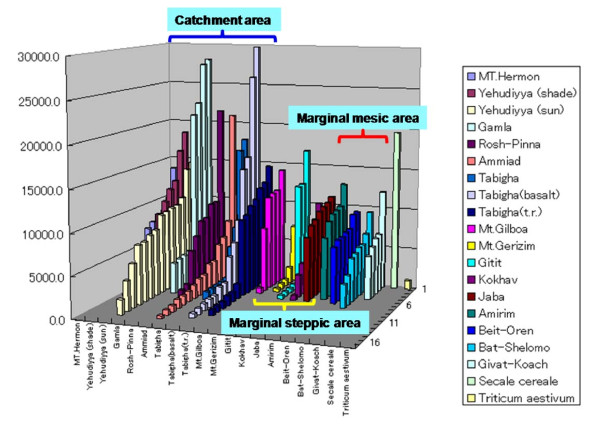
**The copy number of *Revolver *gene in wild emmer wheat *T. dicoccoides *in 18 populations (161 genotypes) representing a wide range of ecologic conditions of soil, temperature, and water availability in Israel**. The populations in the catchment area showed high copy numbers almost over 7,000 per haploid genome and included several genotypes with around 20,000 copies. In contrast, the populations of marginal steppic areas included several genotypes with low copy numbers less than 1,000. In the marginal mesic areas, copy numbers were very stable (from 6,000 to 8,000). This considerable quantitative diversity in the single species and among or in the populations suggests recent mobilization of *Revolver *by ecologic stresses.

Among the *Triticum *species, *Revolver *shows the highest copy number (19,000) in *S. cereale*, and the lowest copy number (2,000) in *T. aestivum *[18]. In this study, the copy numbers of *Revolver *in wild emmer wheat were estimated by slot blot analysis. The *Revolver *cDNA subfamily I (pSc5, 694 bp), which is conserved in *Triticum *species, was used as a probe. Wild emmer wheat, *Triticum dicoccoides *(AABB), is a tetraploid progenitor from which modern tetraploid and hexaploid cultivated wheat is derived. *T. dicoccoides *deserves to be considered as a potential genetic resource for cereal improvement because wild emmer harbors rich genetic diversity for multiple disease resistances, agronomic traits of economic significance, and environmental adaptations [27]. The center of distribution and genetic diversity of *T. dicoccoides *is found in the catchment area of the upper Jordan Valley in Israel and its vicinity [30,31]. We examined the copy number of *Revolver *in wild emmer wheat *T. dicoccoides *in 18 populations (161 genotypes), representing a wide range of ecologic conditions of soil, temperature, and water availability in Israel (Figure 5, Additional files 3 and 4).

All populations were rich in copy number (Additional file 4 and Figure 5). Most populations had a large variance in copy number from around 1,000 to 30,000, despite the predominantly self-fertilizing nature of this species. The populations in the catchment area of Yehudiyya, Gamla, Tabigha, and Rosh-Pinna, where semi-humid and warm climatic conditions prevail, showed high copy numbers, which were almost over 7,000 per haploid genome and included several genotypes having around 20,000 copies (Figure 5). Several populations had higher numbers than 1,100: Yehudiyya shade or sun, Amirim, Bet Oren, Bat Shelomo, Gamla, Giv'at Koah, and J'aba. In the marginal mesic areas of Amirim, Bet Oren, Bat Shelomo, and Giv'at Koah, the copy numbers were very stable: between 6,000 and 8,000. In contrast, the populations of the marginal steppic areas, such as, Mt. Gerizim, Kokhav-Hashahar, and Gitit, included several genotypes with low copy numbers less than 1,000 (Figure 5). This pattern suggests that high *Revolver *copy numbers in northern wild emmer populations appear to be associated with biotic stresses, e.g., pathogens and population densities.

The copy numbers in the populations of Yehudiyya, Ammiad, and Tabigha showed a broad range (between 20,000 and less than 1,000) (Figure 5). Some populations displayed gradual variations from low to high (e.g., Ammiad, Yehudiyya sun and Yehudiyya shade, J'aba, Amirim, Bet Oren, Bat Shelomo, and Giv'at Koah). About half of the populations had polymodal distribution. Some populations were extremely bimodal: Gamla (4 genotypes were from 3,000-4,000 and 4 genotypes were from 20,000-30,000) and Gitit (4 genotypes around 500 and 3 genotypes above 12,000) (Figure 5). The different modes of distributions among the populations suggest differences in *Revolver*'s mobility in each population. In barley, the selective pressures under hot, dry desert conditions significantly correlate with increasing copy numbers of retrotransposon *BARE*-1 [32,33] and microsatellite sequences [34]. *Revolver *showed a variance considerably larger than *BARE*-1. The propagation activity of *Revolver *also might be responsive to both regional and local environmental conditions and its extensive quantitative diversity in the single species *T. dicoccoides *as well as among its local populations at microsites, such as, Yehudiyya and Tabigha, suggest the recent mobilization of *Revolver *by ecological stresses.

Our results show that 6,000 or more copies of *Revolver *exist in the native line from the area that has much precipitation and an average yearly temperature around 20°C; and there was a line that contained as many as 20,000 copies, as does rye wheat. On the other hand, only a few hundred copies existed in the large majority of the lines in the area where the hot-dry monsoon occurred frequently (85 days per year) and the area where the number of sultry nights reached 80 days per year. The remarkable quantitative differences in *Revolver *within the same species growing in different ecosystems illustrate a strong amplification activity within the last 10,000 years.

## Conclusions

Transposon-like factor *Revolver *(US granted patent 7351536), which is 3041 bp in length and includes a 20-bp TIR on either end, is dispersed within the genomes of *Triticum *species. *Revolver *contains an ORF coding for an amino acid sequence of 139 residues that is conserved among *Triticum *species. In *S. cereale*, a 0.7-kb mRNA is actively transcribed from this ORF. Southern blot analysis revealed multiple copies of *Revolver *within *Secale *and *Dasypyrum *species, as well as some copies in diploid species, such as, Einkorn wheat and *Aegilops tauschii*, and in tetraploid species, such as, *Triticum durum*. In contrast, copies were not found in the hexaploid species *T. aestivum*, in which transcribed products were also undetected by northern blot analysis. Through evolution, *Revolver *has been amplified in several *Triticum *species while being lost in others. It is therefore a useful gene for the development of DNA markers for *Triticeae*-related species and for use in *T. aestivum *breeding.

The novel high-copy *Revolver *family is transcriptionally active in rye. Some of the transposon-like elements exist in high copy numbers in the genomes of most eukaryotes, but the great majority of them are inactive, and only a small portion of them retain the ability to transpose [35,36]. Very few transposons have been shown to be transcriptionally active. A copia-like retroelement *BARE*-1 dispersed in 10% of the barley genome [37] is transcribed in somatic tissues [38]. Some LTR retrotransposons such as tobacco Tnt1, Tto1, and *OARE*-1 that are largely inactive, can be transcriptionally activated under conditions of biotic and abiotic stress, including wounding, oxidative stress, and pathogen infection [39,40]. After stress-induced transcription, the rice LTR retrotransposon Tos17 increased in genomic copy number [41]. In maize, a survey of more than 4 × 10^5 ^ESTs identified only 56 retrotransposon cDNAs, supporting the notion that most retrotransposons are inactive [6]. Furthermore, most of these maize sequences are derived from the low-to-middle repetitive LTR retrotransposons and not from the very high copy-number elements that have been responsible for doubling the size of the maize genome in the past 5-6 million years. In humans, only 30-60 L1 elements out of 5 × 10^5 ^comprising 45% of the genome are thought to be active [42]. In general, high-copy retrotransposons show low-level activity except for *BARE*-1. Like *BARE*-1, highly repetitive *Revolver *is transcribed.

A transcriptionally active *Revolver *gene is well conserved among the Triticeae members. The methylated and heterochromatic state of most transposons can cause them to change sequences more rapidly than genes [43,44]. For example, regulation at any stage of the replication cycle for retrotransposons (transcription, translation, reverse transcription, and integration of cDNA elements) can limit transposition. Furthermore, the paucity of maize retrotransposon-derived ESTs indicates that some epigenetic mechanisms might have been repressing the transcription of a large fraction in the genome [15,45,46]. In contrast to these silenced retrotransposons, *Revolver *is transcriptionally active (Figure 1) and might have retained transcriptional activity during the long evolution of Triticeae. The predicted protein encoded by *Revolver *subfamily I includes a c2rf9 motif of a kinase domain and may serve as a transcription factor among this family. The considerable variation in *Revolver *copy numbers among *T. dicoccoides *indicates their propagation activity during the last 10,000 years of *T. dicoccoides *evolution. The name of the novel transposon-like gene *Revolver *means a dynamic factor in constructing genomes through evolution of the Triticeae, associated with ecological stresses, and presumably navigated adaptively by natural selection.

## Methods

### Structural diversities of *Revolver *mRNA

*Revolver *cDNAs were obtained from self-fertile rye, and structural analysis of the cDNA clones was performed. The primers for amplification of *Revolver *cDNA were designed from both ends of a full-length cDNA clone. Total RNA for RT-PCR extracted from seedlings was treated with DNase I. First-strand cDNAs were synthesized by AMV reverse transcriptase (Life Science) with an oligo (dT) primer. Reaction mixtures contained 10 ng of template cDNA, 50 pmoles of each primer (5'-GGCACGAGGGTACGAGTCCGAG-3', 5'-GGCACAACTCATGTAAAAGAGGG-3'), 0.4 mM dNTPs, 1 × LA PCR buffer II, 2.5 mM MgCl_2_, and 0.5 U of *LA Taq *polymerase (Takara) in a volume of 25 μL. The PCR reaction program consisted of 30 cycles of 30 sec at 95°C, 30 sec at 63°C, and 1 min at 72°C. The RT-PCR products were purified, ligated to the pGEM-T vector (Promega), and sequenced.

### *Revolver *Protein

The *Revolver *ORF in cDNA subfamily I (pSc5) was excised by restriction enzyme *Sal *I and *Nco *I and integrated into the plasmid vector pET-32a (+) that had also been digested by *Sal *I and *Nco *I. The plasmid was transformed into *E. coli *BL21 (DE3) cells containing pLysS. The cells were grown in 2YT medium containing carbenicillin and chloramphenicol. The *Revolver *protein was induced by 1 mM IPTG, and the culture was grown at 20°C overnight. Culture at 37°C resulted in production of an inclusion body. The *Revolver *protein fused to thioredoxin and his•tag was purified by nickel chelating affinity chromatography followed by POROS CM chromatography. After treatment with Factor Xa protease, the enzyme was subjected to Superdex 75 gel filtration. The protein structure encoded by *Revolver *was predicted by the Protein Folding Recognition program Robetta (http://robetta.bakerlab.org/).

### Expressed sequence tag homologues of a new class of transposon *Revolver*

In this study, we searched the publicly disclosed EST database of Triticeae for *Revolver *homologues using a default cutoff expectation value of <e^-20^. Nucleotide sequences were compared with sequences in the non-redundant GenBank+EMBL+DDBJ databases with BLASTN homology search software [47]. Sequence alignment was determined with the computer program DNASIS Pro version 2.10 (Hitachi, Tokyo, Japan). A nonrooted phylogenetic tree was constructed with MEGA version 4.0.2 [48]. The neighbor-joining method [49] was conducted with Kimura's 2-parameter distances [50]

### Use of *Revolver *to generate chromosomal markers

The single primer for amplification of *Revolver *genomic DNA was designed from the 3'-flanking region of a typical clone of *Revolver *(*Revolver*-2). Reaction mixtures contained 10 ng of template genomic DNA, 50 pmoles of single primer (5'-GTAGTCGTCAGGAGTCCTCACCA-3'), 0.4 mM dNTPs, 1 × LA PCR buffer II, 2.5 mM MgCl_2_, and 0.5 U of *LA Taq *polymerase (Takara) in a volume of 50 μL. The PCR reaction program consisted of 30 cycles of 30 sec at 95°C, 1 min at 55°C, and 3 min at 72°C. The four types of PCR products (2.3 kb, 2.8 kb, 3.3 kb, and 4.3 kb) amplified from the rye genome and rye chromosome addition wheat genomes were purified, ligated to the pGEM-T vector (Promega), and sequenced.

### Diversity of *Revolver *in wild Emmer wheat of Israel

In this study, we examined the copy number of *Revolver *by slot blot analysis in 161 *T. dicoccoides *genotypes representing 18 populations collected from various locations in Israel, which cover a wide range of ecologic conditions of soil, temperature, altitude, water availability, and abiotic stresses. The collections were from 14 locations in Israel, namely, Mt. Hermon, Yehudiyya, Gamla, Rosh-Pinna, Tabigha, Mt. Gilboa, Mt. Gerizim, Gitit, Kokhav-Hashahar, J'aba, Amirim, Bet Oren, Bat Shelomo, and Giv'at Koah.

Total DNAs isolated from 161 *T. dicoccoides *genotypes were blotted onto Hybond N+ membranes (Amersham). The nonradioactive chemiluminescence method (Gene Images, Amersham) was used for probe labeling, hybridization, and detection of hybridization sites. The *Revolver *cDNA subfamily I (pSc5, 694 bp), which was conserved in *Triticum *species, was used as a probe. The membranes were hybridized at 60°C for 30 min in hybridization buffer and then hybridized with a labeled probe at 60°C overnight. The membranes were washed in SSC, 0.1% SDS at 60°C for 15 min, then in 0.1 × SSC, 0.1% SDS for 15 min, followed by incubation for 60 min at room temperature in a 10% (w/v) blocking agent in an antibody wash buffer. The membrane was then incubated in the presence of an anti-fluorescein antibody-alkaline phosphatase (AP) conjugate. The unbound conjugate was removed by three washes in 0.3% (v/v) Tween 20 in an antibody wash buffer at room temperature. Hybridization sites were detected with the CDP-star detection reagent. Decomposition of the stabilized dioxetane was catalyzed by the probe-bound AP, and exposure of the X-ray film by the emitted light was then recorded with a fluoro-image analyzer (FUJIFILM FLA-5000). The copy number was calculated on the basis of the most approximate function, which Image Gauge software computed from control slot blot hybridization to a series of measured amounts of *Revolver *cDNA (0, 10, 100, 1,000, 5,000, 10,000, 15,000, 20,000, 30, 000, 50,000, and 100,000 copies).

## Authors' contributions

MT conceived and designed the experiments. MT performed the experiments and analyzed the data in Figures 1, 2, 3, 4 and Additional files 1, 2. MT and AO performed the experiments in Figure 5 and Additional file 4. AB and EN analyzed statistically the data in Figure 5 and Additional file 4. EN contributed the materials in Additional file 3. MT wrote the paper. All authors read and approved the final manuscript.

## Supplementary Material

Additional file 1 **Three classes of *Revolver *mRNA**. *Revolver *cDNAs obtained by RT-PCR, total lengths of 665 to 723 bp, were classified into three subfamilies wherein the regions of the second and third exons were almost identical, while the region of the first exon exhibited a low homology of 60% among the families because of duplication or deletion. Repetitive sequence units composed of 8 to 14 bp are present in the same direction in the first exon as seen on the dot plot. Neighbor-joining tree of *Revolver *cDNA sequences in the Triticeae indicated alongside species names showed major three clusters according to the three sub-families; numbers on branches indicate the boot strap values and homologies. Click here for file

Additional file 2 **Sequence alignment between authentic *Revolver *genomic clones from rye IR27 (*Revolver*-3, 5, 6: AB124641, 124643, 124644) and newly isolated *Revolver *genomic clones (*Revolver*-3 on Chr. 6R, *Revolver*-5 on Chr. 1R, *Revolver*-6 on Chr. 5R: AB646252-646254) from each rye Imperial chromosome added in wheat**. Each genomic clone localized on the chromosomes exhibits homology of 96~98% between the genomes of IR27 and Imperial. Click here for file

Additional file 3 **Geographic distribution of 18 tested populations of wild emmer wheat at 15 sites in Israel**. Populations 1-6 were collected from warm, semi-humid environments on the Golan Plateau and near the Sea of Galilee (Yehudiyya, Gamla, Ammiad, Rosh-Pinna, Tabigha, and Mt. Hermon). Populations 7-11 were collected across a wide geographic and marginal steppic area on northern, eastern, and southern borders of wild emmer distribution involving hot, cold, and xeric peripheries (Mt. Gilboa, Mt. Gerizim, Gitit, Kokhav-Hashahar, and J'aba). Populations 12-15 were collected from marginal Mediterranean areas, which are the humid western borders of wild emmer distribution (Amirim, Bet Oren, Bat Shelomo, and Giv'at Koah). Click here for file

Additional file 4 **Copy numbers of *Revolver *and geographic and climatologic data for 18 populations of *Triticum dicoccoides *in Israel**. Click here for file
