# Supplementary material for: Genomic, RNA, and ecological divergences of the Revolver transposon-like multi-gene family in Triticeae
Source: BMC Evol Biol. 2011 Sep 25;11:269. doi: 10.1186/1471-2148-11-269 (PMC3203089; doi:10.1186/1471-2148-11-269)
Supplement: Additional file 1 — Three classes of Revolver mRNA. Revolver cDNAs obtained by RT-PCR, total lengths of 665 to 723 bp, were classified into three subfamilies wherein the regions of the second and third exons were almost identical, while the region of the first exon exhibited a low homology of 60% among the families because of duplication or deletion. Repetitive sequence units composed of 8 to 14 bp are present in the same direction in the first exon as seen on the dot plot. Neighbor-joining tree of Revolver cDNA sequences in the Triticeae indicated alongside species names showed major three clusters according to the three sub-families; numbers on branches indicate the boot strap values and homologies. [file 1471-2148-11-269-S1.PPT]

## Slide 1
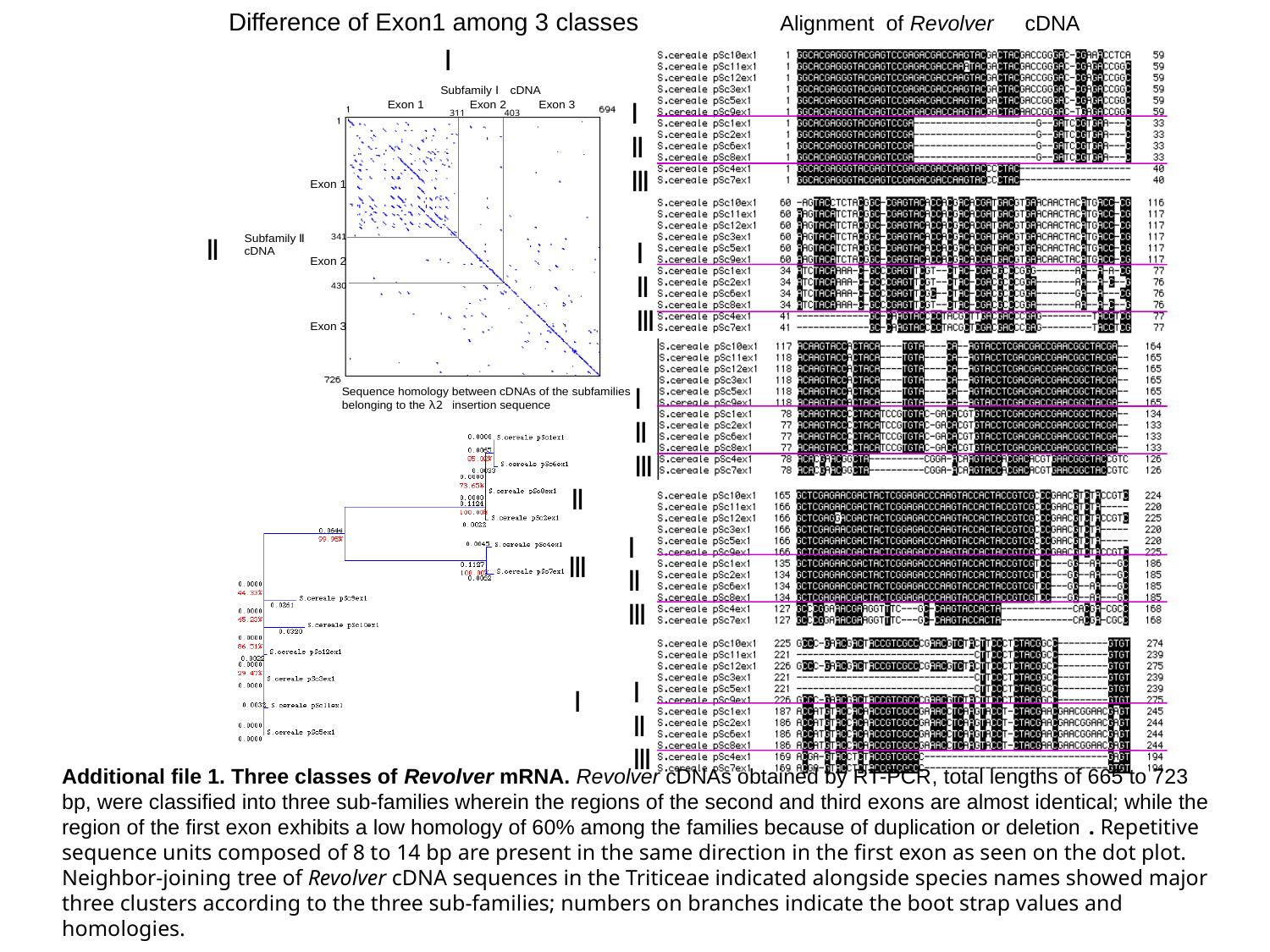

Difference of Exon1 among 3 classes
Alignment of Revolver　cDNA
Ⅰ
Ⅰ
Ⅱ
Ⅲ
Ⅰ
Ⅱ
Ⅲ
Ⅰ
Ⅱ
Ⅲ
Ⅰ
Ⅱ
Ⅲ
Ⅰ
Ⅱ
Ⅲ
Ⅱ
Ⅱ
Ⅲ
 Ⅰ
Additional file 1. Three classes of Revolver mRNA. Revolver cDNAs obtained by RT-PCR, total lengths of 665 to 723 bp, were classified into three sub-families wherein the regions of the second and third exons are almost identical; while the region of the first exon exhibits a low homology of 60% among the families because of duplication or deletion . Repetitive sequence units composed of 8 to 14 bp are present in the same direction in the first exon as seen on the dot plot. Neighbor-joining tree of Revolver cDNA sequences in the Triticeae indicated alongside species names showed major three clusters according to the three sub-families; numbers on branches indicate the boot strap values and homologies.
