# Supplementary material for: Genomic, RNA, and ecological divergences of the Revolver transposon-like multi-gene family in Triticeae
Source: BMC Evol Biol. 2011 Sep 25;11:269. doi: 10.1186/1471-2148-11-269 (PMC3203089; doi:10.1186/1471-2148-11-269)
Supplement: Additional file 2 — Sequence alignment between authentic Revolver genomic clones from rye IR27 (Revolver-3, 5, 6: AB124641, 124643, 124644) and newly isolated Revolver genomic clones (Revolver-3 on Chr. 6R, Revolver-5 on Chr. 1R, Revolver-6 on Chr. 5R: AB646252-646254) from each rye Imperial chromosome added in wheat. Each genomic clone localized on the chromosomes exhibits homology of 96~98% between the genomes of IR27 and Imperial. [file 1471-2148-11-269-S2.PPT]

## Slide 1
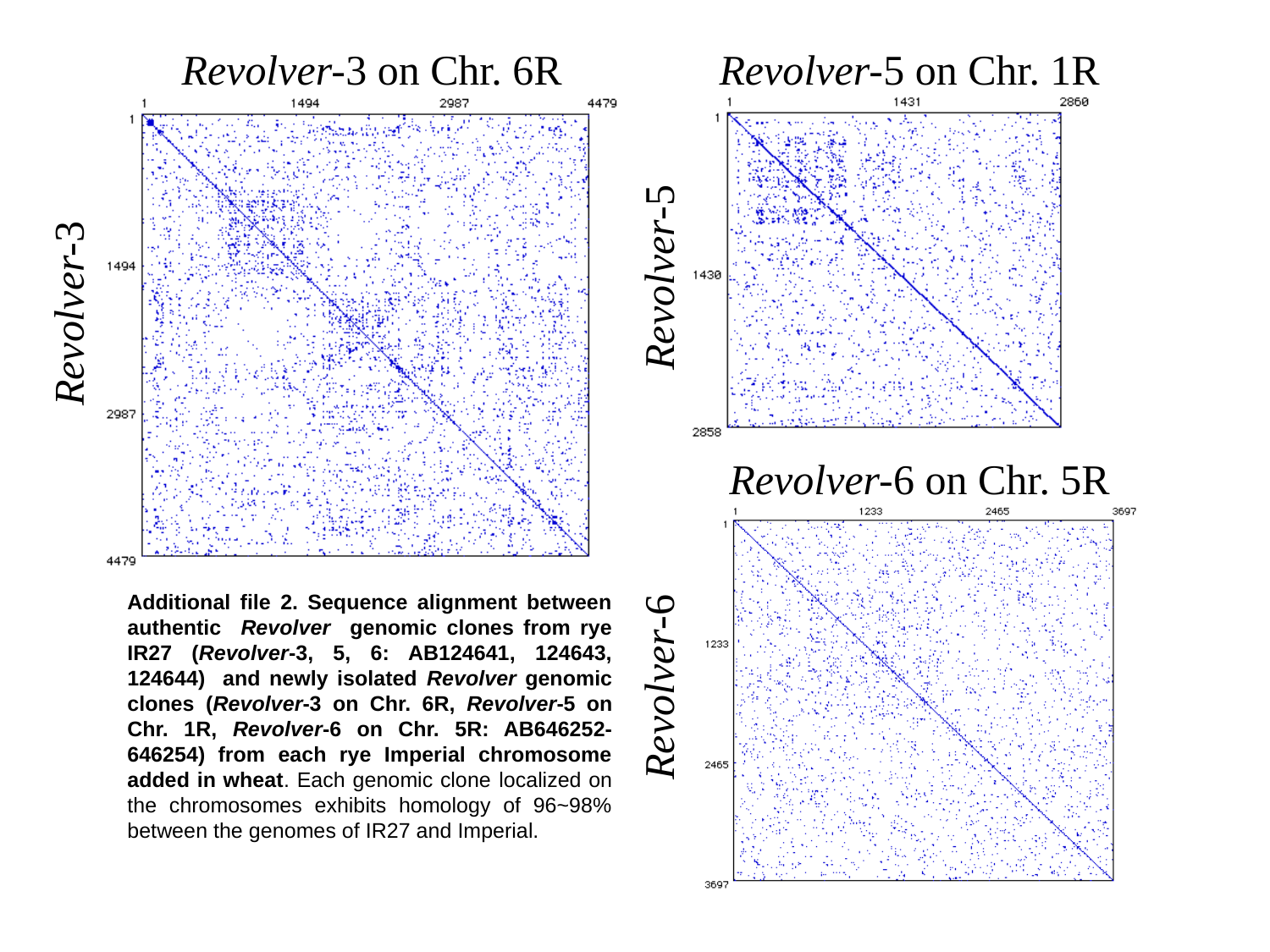

Revolver-3 on Chr. 6R
Revolver-5 on Chr. 1R
Revolver-5
Revolver-3
Revolver-6 on Chr. 5R
Additional file 2. Sequence alignment between authentic Revolver genomic clones from rye IR27 (Revolver-3, 5, 6: AB124641, 124643, 124644) and newly isolated Revolver genomic clones (Revolver-3 on Chr. 6R, Revolver-5 on Chr. 1R, Revolver-6 on Chr. 5R: AB646252-646254) from each rye Imperial chromosome added in wheat. Each genomic clone localized on the chromosomes exhibits homology of 96~98% between the genomes of IR27 and Imperial.
Revolver-6
