# Supplementary material for: Genomic, RNA, and ecological divergences of the Revolver transposon-like multi-gene family in Triticeae
Source: BMC Evol Biol. 2011 Sep 25;11:269. doi: 10.1186/1471-2148-11-269 (PMC3203089; doi:10.1186/1471-2148-11-269)
Supplement: Additional file 3 — Geographic distribution of 18 tested populations of wild emmer wheat at 15 sites in Israel. Populations 1-6 were collected from warm, semi-humid environments on the Golan Plateau and near the Sea of Galilee (Yehudiyya, Gamla, Ammiad, Rosh-Pinna, Tabigha, and Mt. Hermon). Populations 7-11 were collected across a wide geographic and marginal steppic area on northern, eastern, and southern borders of wild emmer distribution involving hot, cold, and xeric peripheries (Mt. Gilboa, Mt. Gerizim, Gitit, Kokhav-Hashahar, and J'aba). Populations 12-15 were collected from marginal Mediterranean areas, which are the humid western borders of wild emmer distribution (Amirim, Bet Oren, Bat Shelomo, and Giv'at Koah). [file 1471-2148-11-269-S3.PPT]

## Slide 1
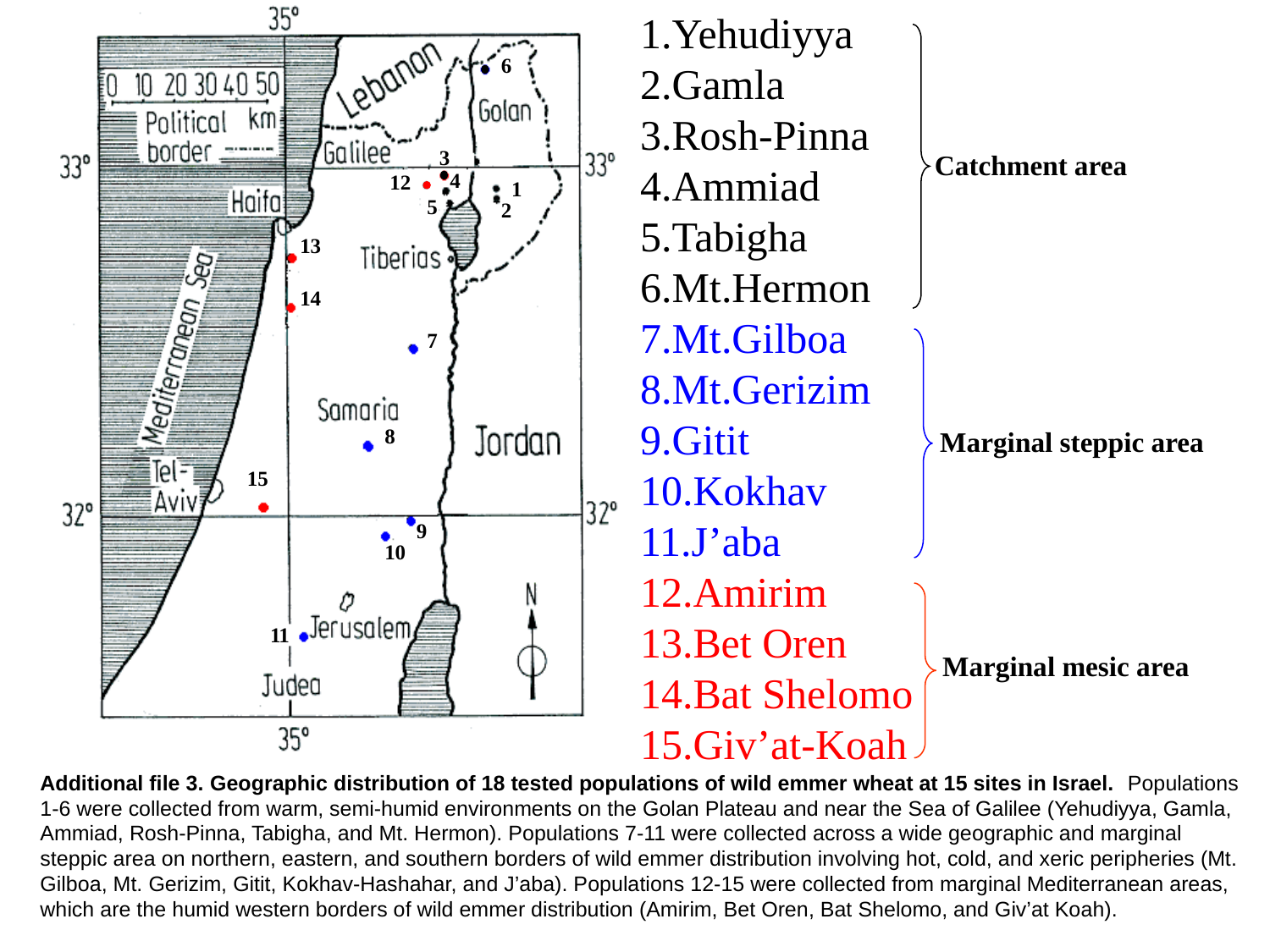

1.Yehudiyya
2.Gamla
3.Rosh-Pinna
4.Ammiad
5.Tabigha
6.Mt.Hermon
7.Mt.Gilboa
8.Mt.Gerizim
9.Gitit
10.Kokhav
11.J’aba
12.Amirim
13.Bet Oren
14.Bat Shelomo
15.Giv’at-Koah
Catchment area
Marginal steppic area
Marginal mesic area
Additional file 3. Geographic distribution of 18 tested populations of wild emmer wheat at 15 sites in Israel. Populations 1-6 were collected from warm, semi-humid environments on the Golan Plateau and near the Sea of Galilee (Yehudiyya, Gamla, Ammiad, Rosh-Pinna, Tabigha, and Mt. Hermon). Populations 7-11 were collected across a wide geographic and marginal steppic area on northern, eastern, and southern borders of wild emmer distribution involving hot, cold, and xeric peripheries (Mt. Gilboa, Mt. Gerizim, Gitit, Kokhav-Hashahar, and J’aba). Populations 12-15 were collected from marginal Mediterranean areas, which are the humid western borders of wild emmer distribution (Amirim, Bet Oren, Bat Shelomo, and Giv’at Koah).
Fig. 4. Geographic distribution of tested 18 populations of wild emmer wheat at 15 sites in Israel. The populations numbered 1-5 were collected from, warm, humid environments on the Golan Plateau and near the Sea of Galilee (Yehudiyya, Gamla, Ammaid, Rosh-Pinna, Tabigha).
The populations numbered 6-11 were collected across a wide geographic and marginal steppic area on northern, eastern, and southern borders of wild emmer distribution, involving hot, cold and xeric peripheries (Mt. Hermon, Mt. Gilboa, Mt. Gerizim, Gitit, Kokhav-Hashahar, J’aba).
The populations numbered 12-15 were collected from Marginal Mediterranean areas, which are the western borders of wild emmer distribution (Amirim, Beit-Oren, Bat-Shelomo, Givat-Koach).
