# Supplementary material for: Genomic, RNA, and ecological divergences of the Revolver transposon-like multi-gene family in Triticeae
Source: BMC Evol Biol. 2011 Sep 25;11:269. doi: 10.1186/1471-2148-11-269 (PMC3203089; doi:10.1186/1471-2148-11-269)
Supplement: Additional file 4 — Copy numbers of Revolver and geographic and climatologic data for 18 populations of Triticum dicoccoides in Israel. [file 1471-2148-11-269-S4.PPT]

## Slide 1
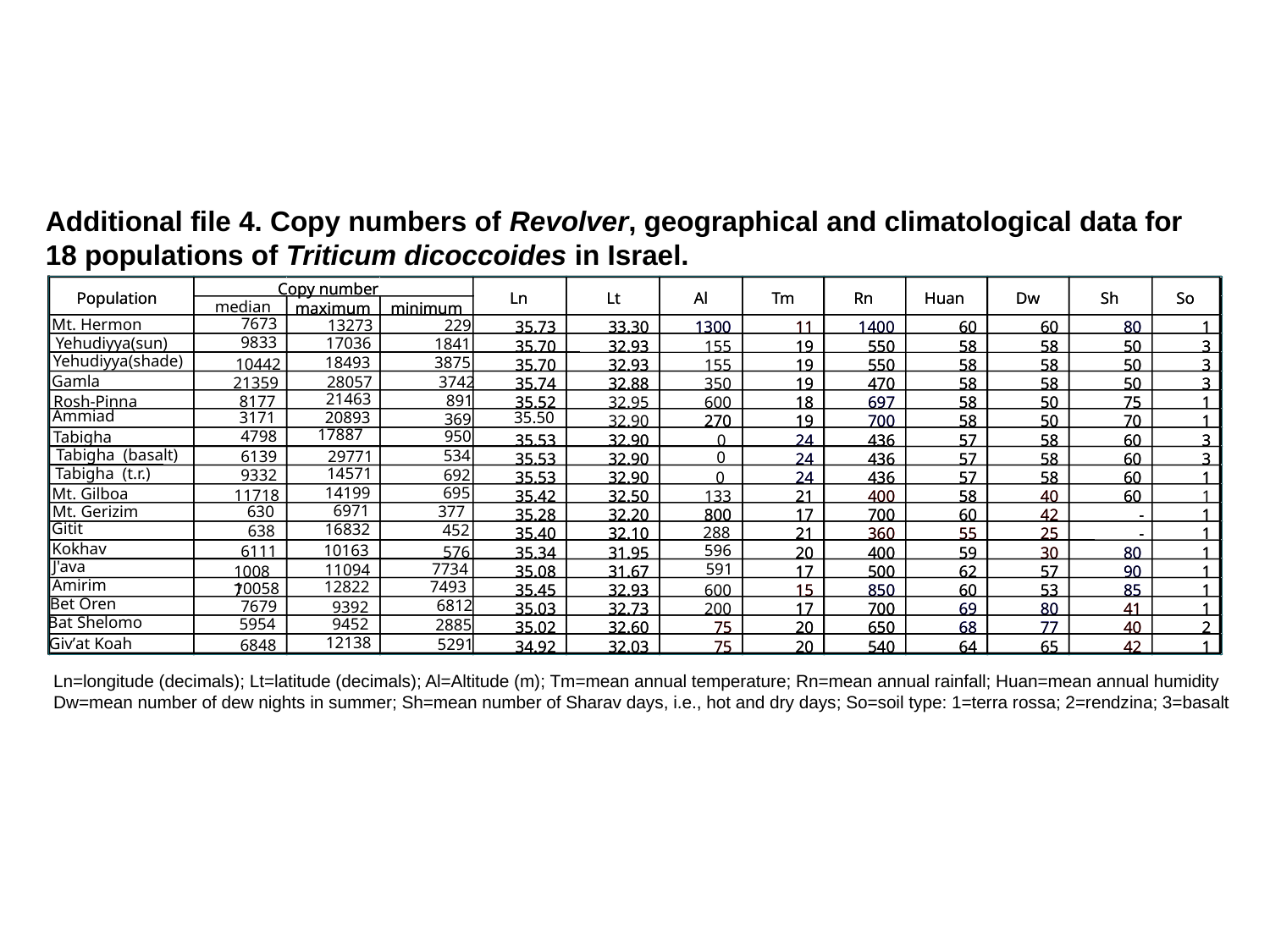

Additional file 4. Copy numbers of Revolver, geographical and climatological data for 18 populations of Triticum dicoccoides in Israel.
 Copy number
 Copy number
Population
Population
Ln
Ln
Lt
Lt
Al
Al
Tm
Tm
Rn
Rn
Huan
Huan
Dw
Dw
Sh
Sh
So
So
median
maximum
maximum
minimum
minimum
7673
Mt. Hermon
13273
229
35.73
35.73
33.30
33.30
1300
1300
11
11
1400
1400
60
60
60
60
80
80
1
1
9833
Yehudiyya(sun)
17036
1841
35.70
35.70
32.93
32.93
155
19
19
550
550
58
58
58
58
50
50
3
3
Yehudiyya(shade)
18493
3875
10442
35.70
35.70
32.93
32.93
155
19
19
550
550
58
58
58
58
50
50
3
3
Gamla
28057
3742
21359
35.74
35.74
32.88
32.88
350
19
19
470
470
58
58
58
58
50
50
3
3
21463
891
Rosh-Pinna
8177
35.52
35.52
32.95
600
18
18
697
697
58
58
50
50
75
75
1
1
Ammiad
3171
35.50
20893
369
32.90
270
270
19
19
700
700
58
58
50
50
70
70
1
1
17887
4798
950
Tabigha
35.53
35.53
32.90
32.90
0
24
24
436
436
57
57
58
58
60
60
3
3
Tabigha (basalt)
534
6139
29771
0
35.53
35.53
32.90
32.90
24
24
436
436
57
57
58
58
60
60
3
3
Tabigha (t.r.)
14571
9332
692
35.53
35.53
32.90
32.90
 0
24
24
436
436
57
57
58
58
60
60
1
1
14199
695
Mt. Gilboa
11718
35.42
35.42
32.50
32.50
133
21
21
400
400
58
58
40
40
60
60
1
6971
Mt. Gerizim
377
 630
35.28
35.28
32.20
32.20
300
800
17
17
700
700
60
60
42
42
-
-
1
1
Gitit
16832
452
 638
288
35.40
35.40
32.10
32.10
21
21
360
360
55
55
25
25
-
-
1
1
Kokhav
10163
596
6111
576
35.34
35.34
31.95
31.95
20
20
400
400
59
59
30
30
80
80
1
1
J'ava
7734
591
11094
10087
35.08
35.08
31.67
31.67
17
17
500
500
62
62
57
57
90
90
1
1
Amirim
12822
7493
10058
35.45
35.45
32.93
32.93
600
15
15
850
850
60
60
53
53
85
85
1
1
Bet Oren
6812
7679
9392
35.03
35.03
32.73
32.73
200
17
17
700
700
69
69
80
80
41
41
1
1
Bat Shelomo
5954
9452
2885
35.02
35.02
32.60
32.60
75
75
20
20
650
650
68
68
77
77
40
40
2
2
12138
Giv’at Koah
5291
6848
34.92
34.92
32.03
32.03
75
75
20
20
540
540
64
64
65
65
42
42
1
1
Ln=longitude (decimals); Lt=latitude (decimals); Al=Altitude (m); Tm=mean annual temperature; Rn=mean annual rainfall; Huan=mean annual humidity
Dw=mean number of dew nights in summer; Sh=mean number of Sharav days, i.e., hot and dry days; So=soil type: 1=terra rossa; 2=rendzina; 3=basalt
